# Supplementary material for: TMK-based cell-surface auxin signalling activates cell-wall acidification
Source: Nature. 2021 Oct 27;599(7884):278–82. doi: 10.1038/s41586-021-03976-4 (PMC8549421; doi:10.1038/s41586-021-03976-4)
Supplement: Supplementary file 1 — Supplementary Tables 2–4 and Supplementary Figs. 1 and 2. [file 41586_2021_3976_MOESM1_ESM.pdf]

nature portfolio

https://doi.org/10.1038/s41586-021-03976-4

Supplementary information

TMK-based cell-surface auxin signalling activates cell-wall acidification

In the format provided by the authors and unedited

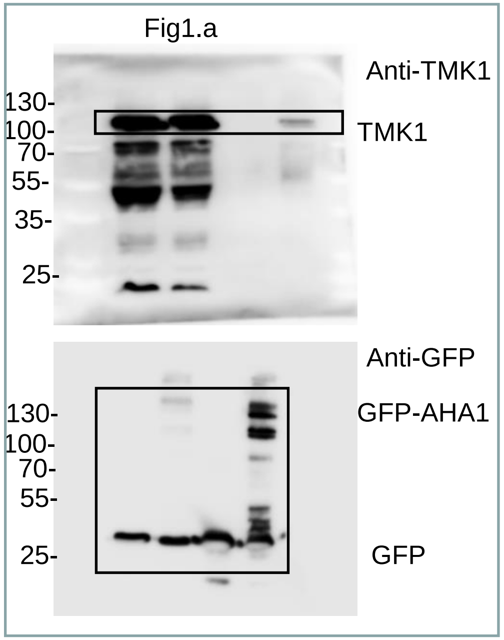
Fig1.a
Anti-TMK1
130-
100-
70-
55-
35-
25-
TMK1
Anti-GFP
130-
100-
70-
55-
25-
GFP-AHA1
GFP

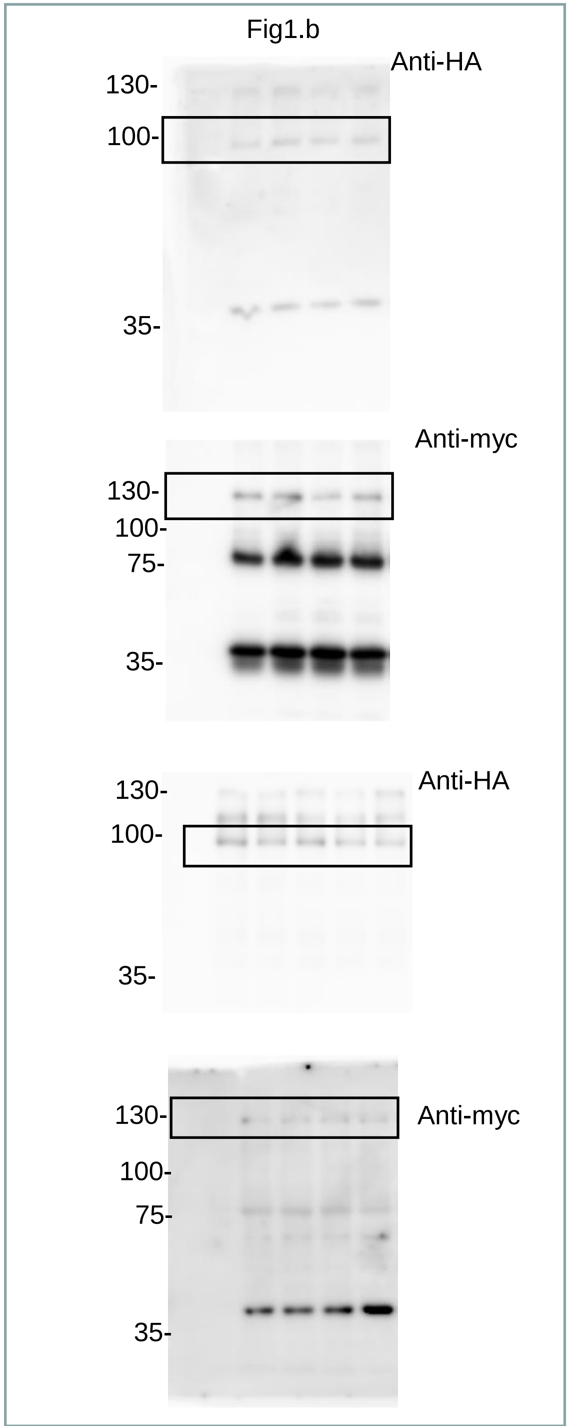
Fig1.b
Anti-HA
130-
100-
35-
Anti-myc
130-
100-
75-
35-
Anti-HA
130-
100-
35-
Anti-myc
130-
100-
75-
35-

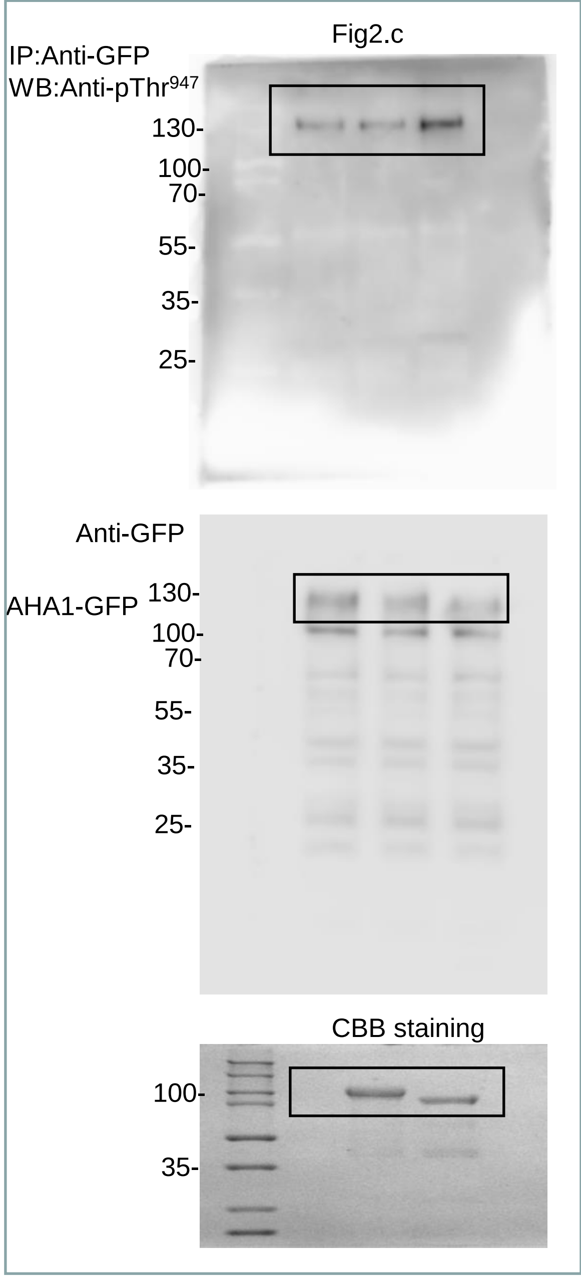
Fig2.c
IP:Anti-GFP
WB:Anti-pThr947
130-
100-
70-
55-
35-
25-
Anti-GFP
AHA1-GFP
130-
100-
70-
55-
35-
25-
CBB staining
100-
35-

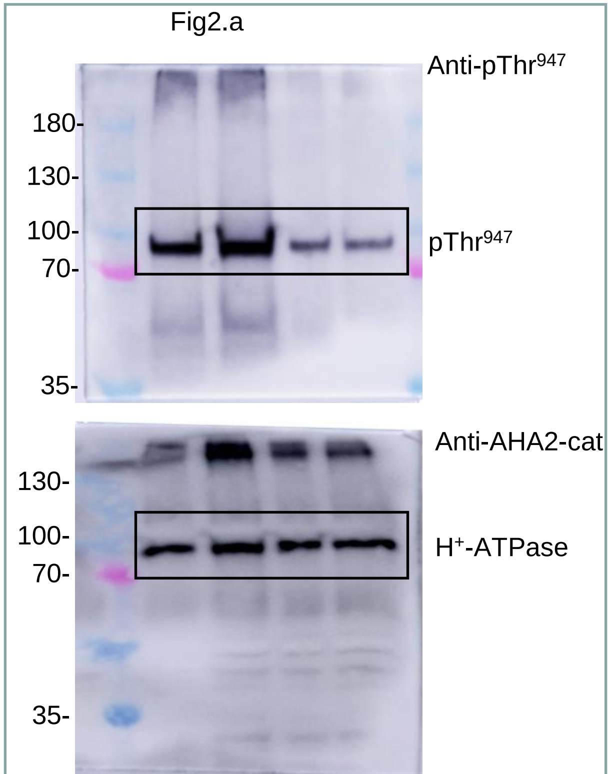
Fig2.a
Anti-pThr947
180-
130-
100-
70-
35-
pThr947
Anti-AHA2-cat
130-
100-
70-
35-
H+-ATPase

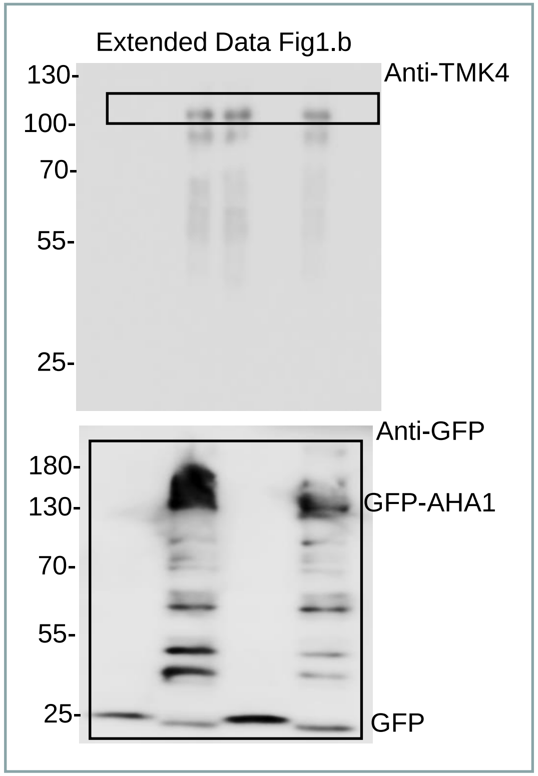
Extended Data Fig1.b
Anti-TMK4
130-
100-
70-
55-
25-
Anti-GFP
180-
130-
GFP-AHA1
70-
55-
25-
GFP

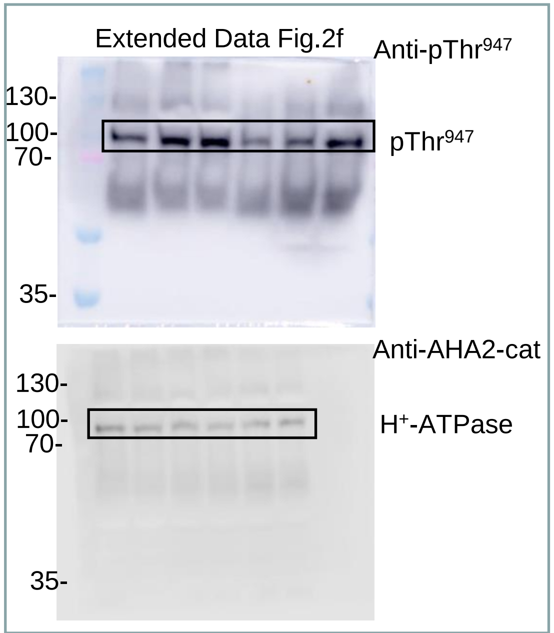
Extended Data Fig.2f
Anti-pThr947
130-
100-
70-
pThr947
35-
Anti-AHA2-cat
130-
100-
70-
H+-ATPase
35-

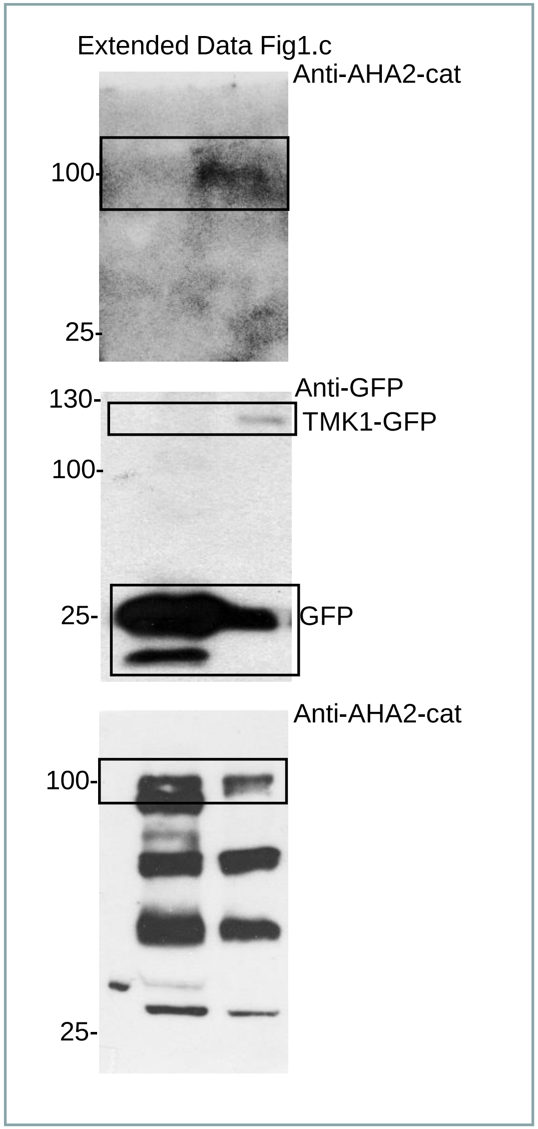
Extended Data Fig1.c
Anti-AHA2-cat
100-
25-
Anti-GFP
130-
TMK1-GFP
100-
25-
GFP
Anti-AHA2-cat
100-
25-

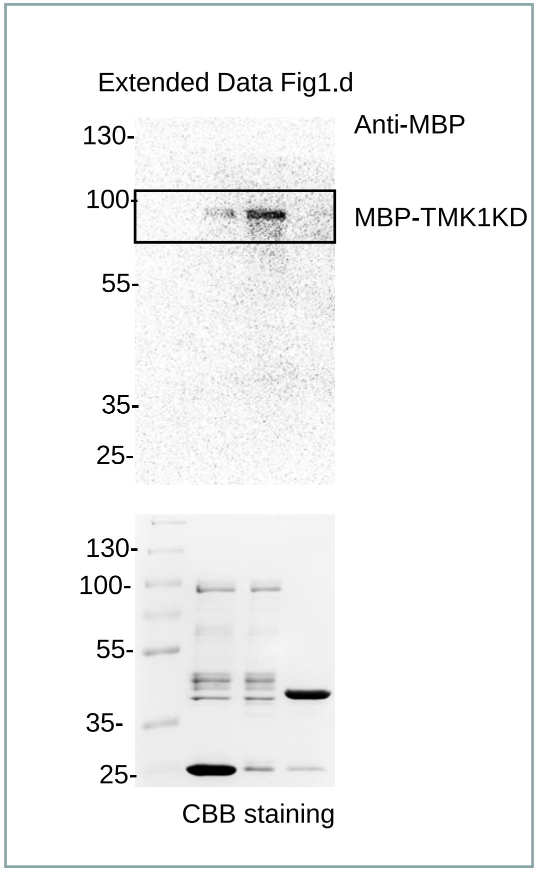
Extended Data Fig1.d
Anti-MBP
130-
100-
MBP-TMK1KD
55-
35-
25-
130-
100-
55-
35-
25-
CBB staining
