# Supplementary material for: TMK-based cell-surface auxin signalling activates cell-wall acidification
Source: Nature. 2021 Oct 27;599(7884):278–82. doi: 10.1038/s41586-021-03976-4 (PMC8549421; doi:10.1038/s41586-021-03976-4)
Supplement: Supplementary file 2 — Reporting Summary [file 41586_2021_3976_MOESM2_ESM.pdf]

nature portfolio

Corresponding author(s): Zhenbiao Yang

Last updated by author(s): Aug 6, 2021

Reporting Summary

Nature Portfolio wishes to improve the reproducibility of the work that we publish. This form provides structure for consistency and transparency in reporting. For further information on Nature Portfolio policies, see our Editorial Policies and the Editorial Policy Checklist.

Statistics

For all statistical analyses, confirm that the following items are present in the figure legend, table legend, main text, or Methods section.

n/a	Confirmed	
☐	☒	The exact sample size (*n*) for each experimental group/condition, given as a discrete number and unit of measurement
☐	☒	A statement on whether measurements were taken from distinct samples or whether the same sample was measured repeatedly
☐	☒	The statistical test(s) used AND whether they are one- or two-sided<br>*Only common tests should be described solely by name; describe more complex techniques in the Methods section.*
☒	☐	A description of all covariates tested
☐	☒	A description of any assumptions or corrections, such as tests of normality and adjustment for multiple comparisons
☐	☒	A full description of the statistical parameters including central tendency (e.g. means) or other basic estimates (e.g. regression coefficient) AND variation (e.g. standard deviation) or associated estimates of uncertainty (e.g. confidence intervals)
☐	☒	For null hypothesis testing, the test statistic (e.g. *F*, *t*, *r*) with confidence intervals, effect sizes, degrees of freedom and *P* value noted<br>*Give P values as exact values whenever suitable.*
☒	☐	For Bayesian analysis, information on the choice of priors and Markov chain Monte Carlo settings
☒	☐	For hierarchical and complex designs, identification of the appropriate level for tests and full reporting of outcomes
☒	☐	Estimates of effect sizes (e.g. Cohen's *d*, Pearson's *r*), indicating how they were calculated

Our web collection on statistics for biologists contains articles on many of the points above.

Software and code

Policy information about availability of computer code

Data collection	Proteomic data was collected by Orbitrap Fusion (Thermo Fisher Scientific, Watham, MA), by software Thermo Xcalibur 3.0.63
Data analysis	1: Arabidopsis root and hypocotyl tissue and cell length are all measured by ImageJ (JAVA 1.8.0_172). 2: FRET analyzer , an ImagJ plug-in, was used to analyze FRET signal. 3: The ratio metric image was analyzed and quantified by Fiji (JAVA 1.8.0_172), using a macro language, which was described in the manuscript. 4:LC-MS data were analyzed with Maxquant (version 1.6.2.6) with Andromeda search engine.

For manuscripts utilizing custom algorithms or software that are central to the research but not yet described in published literature, software must be made available to editors and reviewers. We strongly encourage code deposition in a community repository (e.g. GitHub). See the Nature Portfolio guidelines for submitting code & software for further information.

Data

Policy information about availability of data

All manuscripts must include a data availability statement. This statement should provide the following information, where applicable:

- Accession codes, unique identifiers, or web links for publicly available datasets
- A description of any restrictions on data availability
- For clinical datasets or third party data, please ensure that the statement adheres to our policy

Code and Data availability statement was included in Methods Section. Mass spectrometry raw data associated with Figure 2e, extended Data Figure 2a,b are available at the MassIVE under accession number: MSV000087822.Source Data (gel and graphs) are provided with manuscript. The data supporting the findings in this study are available and described within the manuscript and extend data information file.

Field-specific reporting

Please select the one below that is the best fit for your research. If you are not sure, read the appropriate sections before making your selection.

☒ Life sciences ☐ Behavioural & social sciences ☐ Ecological, evolutionary & environmental sciences

For a reference copy of the document with all sections, see nature.com/documents/nr-reporting-summary-flat.pdf

Life sciences study design

All studies must disclose on these points even when the disclosure is negative.

Sample size	Sample size calculation was not performed. We determined the number of samples in each experiment as commonly accepted standards in the field. 1: Western blot. All the western blot assays were repeat 3-4 times and the intensity peak of the target bands were measured by ImageJ. 2: Quantitative analysis of hypocotyl elongation zone apoplastic pH value. Each data set was from the measurement of 10-20 etiolated hypocotyls of different genotypes. 3: MS to identified in vitro peptides phosphorylation. Two biological repeats were included, in each biological repeat, 3 independent technique repeats were included. 4: TMT-label based phosphoproteomics MS. 0.45 mg proteins of each sample were applied to TMT-labeled and ms analysis. 2 independent biological repeats were performed for this assay. 1-5 mg proteins were applied to IP-MS. 5: For FRET assay. The data was collected and analyzed from 10 individual cells. The experiment was repeated 3 times.
Data exclusions	No data were excluded from this study.
Replication	The data in thsi paper is highly replicable, as the companion paper conduct several experiments independently used same materials produced same results. All the measures in this study were conducted in 2-4 times biology repeats, which start from germination of the seedlings. Each set of the data were collected and analyzed independently.
Randomization	The study does not involved work that required random allocation. The sample were allocated into experimental groups based on their genotypes, for instance, by wild type or specific gene mutations. The randomization was not applied in this study.
Blinding	No double blinding is applied in this study. For this current study, blinding is not relevant.

Reporting for specific materials, systems and methods

We require information from authors about some types of materials, experimental systems and methods used in many studies. Here, indicate whether each material, system or method listed is relevant to your study. If you are not sure if a list item applies to your research, read the appropriate section before selecting a response.

Materials & experimental systems

n/a	Involved in the study	
☐	☒	Antibodies
☒	☐	Eukaryotic cell lines
☒	☐	Palaeontology and archaeology
☒	☐	Animals and other organisms
☒	☐	Human research participants
☒	☐	Clinical data
☒	☐	Dual use research of concern

Methods

n/a	Involved in the study	
☒	☐	ChIP-seq
☒	☐	Flow cytometry
☒	☐	MRI-based neuroimaging

Antibodies

Antibodies used	1: The anti-HA (Invitrogen, # 26183, 1:2000 dilution), GFP (Chromotek, #3h9, 1:1000 dilution) , Myc (sinobiological, #100029-MM08, 1:1000 dilution), GST (Santa Cruz, #sc-138, 1:1000 dilution) and MBP (Invitrogen, PA1-989, 1:1000 dilution) antibodies that were used in this study are all commercial available with the validations.<br>2: pT947 AHA antibody was described in manuscript, which was generated from rabbit (1:5000 dilution).
Validation	Validation statements of commercial primary antibodies are available from manufacturers. α-GFP (https://www.chromotek.com/fileadmin/content/PDFs/Data_Sheets/3h9_Datasheet_GFP_antibody__3H9.pdf), α-HA-HRP (https://www.thermofisher.com/order/genome-database/dataSheetPdf producttype=antibody&productsubtype=antibody_primary&productId=26183-HRP&version=133), α-myc (http://www.sinobiologicalcdn.com/reagent/100029-MM08.pdf). α-MBP (https://www.thermofisher.com/order/genome-database/dataSheetPdf?producttype=antibody&productsubtype=antibody_primary&productId=PA1-989&version=133).α-GST (https://datasheets.scbt.com/sc-138.pdf). pT947 antibody was validated as reference: doi:10.1093/pcp/pcq078.
